# Multiparametric cardiac magnetic resonance identifies macrophage nitric oxide synthase 2-mediated benefits of preventive sodium-glucose cotransporter 2 inhibition in a mouse model of metabolic heart disease

**DOI:** 10.1016/j.jocmr.2025.101972

**Published:** 2025-10-10

**Authors:** Julia E. Bresticker, Caitlin M. Pavelec, Thomas P. Skacel, John T. Echols, R. Jack Roy, Leigh A. Bradley, Edgar H. Macal, Brent A. French, André Marette, Christopher M. Kramer, Brant E. Isakson, Amit R. Patel, Matthew J. Wolf, Frederick H. Epstein

**Affiliations:** a Department of Biomedical Engineering, University of Virginia, Charlottesville, Virginia, USA; b Division of Cardiovascular Medicine, University of Virginia, Charlottesville, Virginia, USA; c The Robert M. Berne Cardiovascular Research Center, University of Virginia, Charlottesville, Virginia, USA; d Department of Molecular Physiology and Biological Physics, University of Virginia, Charlottesville, Virginia, USA; e Department of Medicine, Québec Heart and Lung Institute, Université Laval, Québec, Canada

**Keywords:** NOS2, Epicardial adipose tissue, SGLT2 inhibitors, CMR, Metabolic heart disease, Coronary microvascular disease

## Abstract

**Background:**

Sodium-glucose cotransporter 2 (SGLT2) inhibitors improve metabolic and cardiovascular outcomes, but the mechanisms remain incompletely understood. We utilized cardiovascular magnetic resonance (CMR) and complementary methods to investigate whether preventive SGLT2 inhibitor administration attenuates the development of metabolic heart disease in a high-fat, high-sucrose diet (HFHSD) mouse model.

**Methods:**

Male wild-type (WT) C57BL/6 J mice were fed an HFHSD for 18 weeks to induce obesity, coronary microvascular disease, and diastolic dysfunction. WT mice treated preventively with an SGLT2 inhibitor, empagliflozin (EMPA), were compared to untreated WT mice, and mice fed either an HFHSD or standard chow diet with myeloid cell-specific knockout of the *Nos2* gene (*Nos2*^LysMCre^) were compared to floxed controls (*Nos2*^fl/fl^). CMR assessed epicardial adipose tissue (EAT) volume, fatty acid composition (FAC), proton density fat fraction (PDFF), and T1, and myocardial perfusion, and strain. EAT FAC, PDFF, and T1 were quantified using an inversion-recovery multi-echo gradient-echo sequence and a multi-resonance triglyceride model. EAT volume was quantified using cine images. Myocardial perfusion reserve (MPR) and strain were measured using arterial spin labeling, and displacement encoding with stimulated echoes (DENSE), respectively. Histology and flow cytometry assessed EAT remodeling and macrophage polarization.

**Results:**

EMPA treatment reduced EAT volume (0.36 ± 0.18 µL/g vs 0.61 ± 0.25 µL/g, p<0.01) and saturated fatty acid fraction (38.81 [32.83–47.71]% vs 48.06 [43.82–52.65]%, p<0.05), increased EAT T1 (0.799 [0.764–0.859] s vs 0.755 [0.678–0.772] s, p<0.05), and decreased EAT NOS2^+^ macrophages (34.74 [21.38–42.098^]^% vs 46.36 [38.08–61.30]%, p<0.05) compared to controls. EMPA improved diastolic strain rate (2.96 [2.61–3.99] s^-1^ vs 1.68 [1.21–2.80] s^-1^, p<0.01) and adenosine MPR (2.00 ± 0.54 vs 1.37 ± 0.40, p<0.01) compared to controls. Myeloid cell NOS2 knockout mice fed an HFHSD exhibited improved adenosine MPR (1.90 ± 0.47 vs 1.39 ± 0.38, p<0.01) compared to floxed controls.

**Conclusions:**

In this obesity-related metabolic heart disease model, EMPA treatment prevents cardiometabolic dysfunction by improving EAT quantity and quality, coronary microvascular function, and diastolic function. These benefits are mediated in part through macrophage NOS2.

## Graphical abstract

**Figure:**
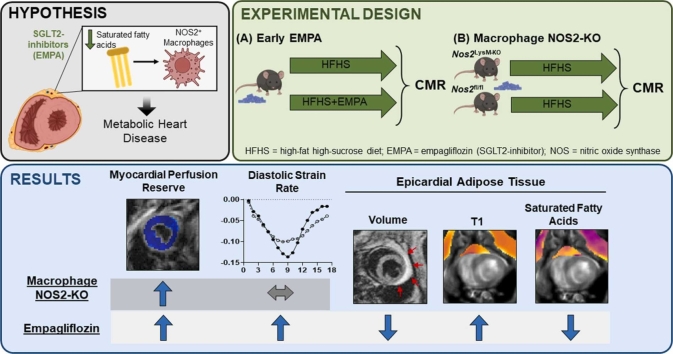

## 1 Introduction

A chronic systemic inflammatory state associated with the accumulation of excess visceral adipose tissue (VAT) is central to the pathogenesis of obesity-related metabolic heart disease (MHD), which is now understood to be a major precursor to heart failure with preserved ejection fraction (HFpEF) [1]. In mouse models of diet-induced obesity, inducible nitric oxide synthase (NOS2, formerly known as iNOS) has been shown to play a critical role in mediating oxidative and nitrosative stress and the related cardiovascular dysfunction [2], [3]. Among visceral fat depots, epicardial adipose tissue (EAT) in particular promotes myocardial inflammation due to its anatomical proximity, shared microcirculation, and potential to infiltrate the myocardium [4]. In obesity, EAT undergoes adipocyte hypertrophy and becomes enriched with proinflammatory saturated fatty acids, initiating an inflammatory cascade marked by cytokine secretion, immune cell recruitment, and proinflammatory macrophage polarization [5], [6], [7]. Cytokines can be trafficked to the myocardium via paracrine and vasocrine pathways, leading to coronary microvascular and diastolic dysfunction—key features of obesity-induced MHD. Despite advances in understanding EAT and key inflammatory mediators such as NOS2 in obesity-induced MHD, critical gaps remain, such as clarifying specific cell types responsible for NOS2 effects. Also, new noninvasive cardiovascular magnetic resonance (CMR) biomarkers to characterize proinflammatory EAT could help elucidate mechanisms of disease progression and therapy.

Sodium-glucose cotransporter 2 (SGLT2) inhibitors have emerged as a promising therapy for patients with obesity-related MHD and HFpEF [8], [9]. Although SGLT2 inhibitors have been shown to reduce EAT volume, suppress adipose tissue inflammation, and improve left ventricular (LV) diastolic function, their mechanisms of cardiovascular protection are not fully understood [10], [11], [12]. The effect of SGLT2 inhibition on the coronary microvascular response to adenosine receptor agonism and associated myocardial perfusion reserve (MPR) have yet to be definitively investigated. Moreover, it is unclear whether SGLT2 inhibitors can modulate proinflammatory EAT changes induced by a high-fat, high-sucrose diet (HFHSD), such as altered fatty acid composition (FAC), adipocyte hypertrophy, or macrophage infiltration and polarization.

The purpose of the present study was to use multiparametric CMR and other methods applied to an HFHSD mouse model of obesity-induced MHD to investigate the effects of preventive SGLT2 inhibition on EAT quantity and quality, macrophages, coronary microvascular dysfunction, and diastolic dysfunction. The HFHSD mouse model recapitulates features of obesity-induced MHD, including obesity, glucose intolerance, VAT accumulation, oxidative stress, coronary microvascular dysfunction, and diastolic dysfunction [3], [13], [14]. Also, using a Cre-Lox system to knockout the *Nos2* gene in myeloid cells, we tested the hypothesis that NOS2 derived specifically from macrophages contributes to impaired coronary microvascular and diastolic dysfunction in the HFHSD model.

## 2 Methods

### 2.1 Experimental design

All animal studies were performed in accordance with protocols that conformed to the Declaration of Helsinki as well as the Guide for Care and Use of Laboratory Animals [15] and were approved by the Animal Care and Use Committee at the University of Virginia. All mice were maintained at the University of Virginia Center for Comparative Medicine pathogen-free vivarium facility. Male mice were used in this study because the time course and degree of EAT accumulation and coronary microvascular and diastolic dysfunction due to an HFHSD have been established [3], [14]. Experiments were performed to test the following hypotheses: (1) that preventive treatment with an SGLT2 inhibitor given at the initiation of an HFHSD reduces EAT accumulation, modifies EAT quality, improves MPR, improves diastolic dysfunction, and reduces NOS2^+^ M1 macrophages in the EAT and heart, and (2) that macrophages are the NOS2-expressing cell type responsible for the development of coronary microvascular and diastolic dysfunction resulting from 18 weeks of an HFHSD.

#### 2.1.1 Effect of preventive SGLT2 inhibition on obesity-induced metabolic heart disease

**Fig. 1:**
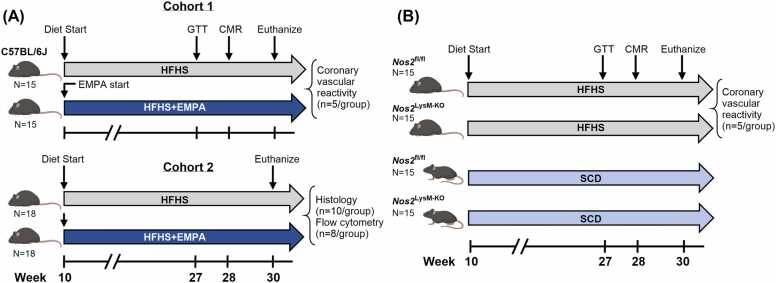
Experimental protocols. (A) Experimental protocol for EMPA treatment. In cohort one, ten-week-old C57BL/6 J male mice were fed an HFHS diet with or without EMPA (15/group). At 27 weeks of age (17 weeks on diet), mice underwent GTT, followed by CMR at 28 weeks (18 weeks on diet). Mice were euthanized at 30 weeks and coronary microvessels were harvested from a subset of mice to assess ex-vivo vascular reactivity (5/group). Cohort two was designated for post-mortem analysis. The timeline for cohort two was the same as that of cohort one. Mice were euthanized at 30 weeks for tissue collection and analysis, including macrophage flow cytometry (8/group) and adipose tissue and myocardial histology (10/group). (B) Experimental protocol for investigating the role of macrophage NOS2. Ten-week-old *Nos2*^fl/fl^ or *Nos2*^LysM-KO^ male mice were fed an HFHS diet or an SCD (15/group). At 27 weeks (17 weeks on diet), mice underwent GTT, followed by CMR at 28 weeks (18 weeks on diet). Mice were euthanized at 30 weeks of age and coronary microvessels were harvested from a subset of HFHS-fed mice to assess ex-vivo vascular reactivity (5/group). *EMPA* empagliflozin, *HFHS* high-fat high-sucrose, *GTT* glucose tolerance testing, *CMR* cardiovascularc magnetic resonance, *NOS2* inducible nitric oxide synthase, *SCD* standard chow diet

Two cohorts of mice were studied to assess the effects of SGLT2 inhibition on obesity-induced MHD (Fig. 1A). In the first cohort, wild-type (WT) male C57BL/6 J mice (Jackson Laboratories, strain #000664) were randomized into the following two groups (n = 15/group): (1) mice fed an HFHSD (40% (1620/4052) kcal fat, 40% (1620/4052) kcal sucrose; Diet 123727, Research Diets Inc) (HFHS), and (2) mice fed an HFHSD with 30 mg/kg/day of the SGLT2 inhibitor, empagliflozin (EMPA), added to the diet (40% (1620/4052) kcal fat, 40% (1620/4052) kcal sucrose; Diet 21011406, Research Diets Inc) (HFHS+EMPA). Empagliflozin has been extensively studied in both preclinical [10], [16] and clinical [8] trials and is recognized as a potent SGLT2 inhibitor with the highest selectivity among agents in its class [17]. Either diet was initiated at 10 weeks of age and was continued for 18 weeks. Glucose tolerance tests (GTT) were performed 17 weeks post-diet (n = 15/group). All mice underwent CMR at 18 weeks post-diet (n = 15/group). A subset of 5 mice per group from this cohort was used for coronary arteriolar reactivity experiments following euthanasia at 20 weeks on diet.

A second, independent cohort of mice was designated for additional post-mortem analyses. In this cohort, ten mice per group were used for adipose tissue and myocardial histology and eight mice per group were used for macrophage flow cytometry. No individual mouse underwent more than one type of post-mortem analysis.

#### 2.1.2 Role of macrophage NOS2 in obesity-induced MHD

Male *Nos2* LysM-Cre (*Nos2*^LysM-KO^) mice and homozygous *Nos2* floxed (*Nos2*^fl/fl^) control mice were studied. *Nos2*^LysM-KO^ mice were generated using the Cre-lox system by breeding *Nos2*^fl/fl^ mice with LysM-Cre mice. Genotyping was performed by Transnetyx (Cordova, TN) to confirm the presence of the LysM-Cre transgene and *Nos2* floxed alleles. Four groups of mice were studied (n = 15/group, Fig. 1B) as follows: (1) *Nos2*^fl/fl^ mice fed a standard chow diet (SCD) (SCD-*Nos2*^fl/fl^), (2) *Nos2*^fl/fl^ mice fed an HFHSD (HFHSD-*Nos2*^fl/fl^), (3) *Nos2*^LysM-KO^ mice fed an SCD (SCD-*Nos2*^LysM-KO^), and (4) *Nos2*^LysM-KO^ mice fed an HFHSD (HFHSD-*Nos2*^LysM-KO^). Diets were initiated at 10 weeks of age and were continued for 18 weeks. GTT was performed at 17 weeks post-diet (n = 9/group). All mice underwent CMR at 18 weeks post-diet (n = 15/group). After 20 weeks on diet, a subset of mice from HFHSD-fed groups (n = 5/group) were euthanized and used for coronary arteriolar reactivity experiments.

### 2.2 CMR protocol

CMR studies were performed over two sessions separated by 2–3 days. CMR was performed with either a 9.4T system (Biospec 94/20, Bruker Biospin, Billerica, Massachusetts) or a 7T system (Clinscan, Bruker/Siemens) using a ^1^H transmit-receive quadrature volume radiofrequency coil with 35 mm inner diameter (Bruker BioSpin). During CMR studies, the electrocardiogram (ECG), body temperature, and respiration were continuously monitored (SA Instruments, Stony Brook, New York). Mice were anesthetized with 1% isoflurane and maintained at a body temperature of 36 ± 0.5 °C using circulating warm water.

For all experiments, the CMR protocol included (1) arterial spin labeling (ASL) at rest and with adenosine vasodilation to quantify myocardial blood flow (MBF) and MPR for the assessment of coronary microvascular function [18], [19], [20], (2) displacement encoding with stimulated echoes (DENSE) imaging to measure global longitudinal strain and peak diastolic strain rate (PDSR) for the evaluation of LV systolic and diastolic function [21], [22], and (3) cine imaging covering the entire LV to measure cardiac structure and function parameters including LV mass, end-diastolic wall thickness (EDWT), end-systolic wall thickness (ESWT), end-diastolic volume (EDV), end-systolic volume (ESV), and ejection fraction (EF) [23]. For the experiments investigating SGLT2 inhibition, the CMR protocol also included FAC and T1 mapping to quantify metrics of EAT quality (proton density fat fraction (PDFF), saturated fatty acid fraction (SFA), monounsaturated fatty acid fraction (MUFA), polyunsaturated fatty acid fraction (PUFA), and longitudinal relaxation time, T1) and myocardial PDFF [14], [24], [25]. FAC, EAT T1 mapping and rest and stress ASL were performed at session one of CMR at 9.4T (total scan time approximately 60 min), and cine and DENSE imaging were performed at session two of CMR at 9.4T (total scan time approximately 45 min). For the experiments investigating the role of macrophage NOS2, rest and stress ASL and cine imaging were performed at session one of CMR at 9.4T (total scan time approximately 60 min), and DENSE was performed at session two of CMR at 7T (total scan time approximately 30 min). Body weight was recorded for all mice at the beginning of each imaging study. One HFHS mouse from cohort one of the SGLT2 inhibition study died during CMR and was excluded from all analyses.

The 7T system was used exclusively for DENSE in the macrophage NOS2 experiments, as DENSE had not yet been implemented on the 9.4T scanner at the time those studies were performed. All other CMR measurements were acquired on the 9.4T system. No comparisons of strain or strain rate were made across field strengths or between experimental groups using different scanners.

#### 2.2.1 EAT fatty acid composition and T1 mapping

Joint FAC and T1 mapping was performed using a previously validated ECG-gated inversion-recovery multi-echo gradient-echo sequence [25]. Briefly, the sequence acquired images across multiple inversion times (TIs) and echo times (TEs) to enable simultaneous estimation of water and fat signals as well as T1. A golden-angle radial sampling scheme was employed in-plane and across both the TE and TI dimensions to achieve incoherent undersampling. Imaging parameters included: number of TEs: 20; TE_1_: 1.4 ms; ΔTE = 0.2 ms; number of TIs 30; TI_1_: 3.7 ms; ΔTI = RR-interval; flip angle: 15°; slice thickness: 1.0 mm; number of radial spokes: 21; field of view (FOV): 25 × 25 mm^2^; matrix size: 128 × 128; resolution = 0.2 × 0.2 mm^2^; total scan time approximately 20 min.

#### 2.2.2 Cine imaging

Six to 8 short-axis slices were acquired, covering the LV from base to apex. Imaging parameters included: repetition time (TR): 4.0 ms; TE: 1.35 ms; temporal resolution: 4.0 ms; FOV: 25 × 25 mm^2^; matrix size: 128 × 128; flip angle: 15°; number of averages = 3; resolution = 0.2 × 0.2 mm^2^; total scan time approximately 2 min per slice.

#### 2.2.3 Myocardial perfusion imaging

At session 1, an intraperitoneal catheter was inserted for delivery of the vasodilator, adenosine (Sigma-Aldrich) (18 µg/min), during imaging. Rest perfusion imaging of a mid-ventricular short-axis slice was then performed using a respiratory-gated ASL method [19]. Thereafter, adenosine was infused via indwelling catheter and 10 min later ASL was repeated. Imaging parameters for ASL included: TE: 2.5 ms; TR: 10.0 ms; FOV: 25 × 25 mm^2^; matrix size: 128 × 128; flip angle: 7°; slice thickness: 1.0 mm; saturation band thickness: 2.5 mm; number of averages: 9; resolution = 0.2 × 0.2 mm^2^; total scan time approximately 10 min per rest or stress scan.

#### 2.2.4 Myocardial strain imaging

DENSE strain imaging [21] was performed on a 4-chamber long-axis slice. Imaging parameters included: FOV: 32 × 32 mm^2^; matrix size: 128 × 128; slice thickness: 1.0 mm; TR: 7.0 ms; TE: 2.45 ms; number of averages: 4; spatial resolution = 0.25 × 0.25 mm^2^; total scan time approximately 14 min.

### 2.3 CMR image analysis

Image analysis was performed using MATLAB 2023a (The MathWorks, Natick, Massachusetts). One author was aware of group assignments during CMR and image analysis, and a separate author was aware of group allocations during post-mortem experimentation. For MBF quantification, rest and adenosine-stress perfusion images were analyzed using methods previously described [19]. MPR was calculated as the ratio of stress perfusion to rest perfusion. Strain analysis of DENSE images was performed using the DENSE analysis tool [26], [27]. Global longitudinal strain and PDSR were measured as metrics of systolic and diastolic function, respectively. Cine images were analyzed using Segment version 4.1.0.1 R14284b package. Specifically, the end-diastolic and end-systolic frames were identified, and the endocardial and epicardial contours were manually drawn on these frames for all slices. Using Segment, EDWT, ESWT, EDV, ESV, EF, and LV mass were calculated. Cine images were also used to quantify EAT volume index, defined as EAT volume in microliters divided by the mouse body weight in grams. The EAT was manually segmented at end-diastole for all slices and the combined EAT volume across all slices was calculated as the number of pixels × 0.038 mm^3^/pixel.

For EAT FAC, PDFF, and T1 quantification, incoherently undersampled inversion-recovery multi-echo images were reconstructed using a higher-order singular value decomposition method, as previously described [25]. Parametric maps of PDFF, T1, and individual fatty acid components (SFA, MUFA, PUFA) were generated from multi-TI and multi-echo images. Briefly, a multi-resonance triglyceride signal model derived from a mean triglyceride spectrum was used to describe the complex MR signal [24], [28], [29]. The fat signal was modeled as a sum of nine chemically distinct resonances, each with a known chemical shift and relative amplitude [30]. The complex signal at each voxel was fit across multiple echo and inversion times to jointly estimate water and fat amplitudes and T1. The relative amplitudes of the nine fat peaks were used to estimate the number of double bonds and methylene-interrupted double bonds per triglyceride. These parameters, along with a fixed estimated chain length, were then used to calculate the relative proportions of saturated, monounsaturated, and polyunsaturated fatty acids in each voxel using established spectral relationships [24]. Finally, PDFF was calculated as the ratio of the total fat signal to the sum of the water and fat signals within each voxel.

Manual segmentation of EAT and subcutaneous adipose tissue (SAT) was performed on fat-only images by a single trained operator with 3 years of experience in cardiac MRI and adipose tissue segmentation. The EAT depot was defined as the adipose tissue directly surrounding the right and left ventricles, and the SAT depot was defined as the entire adipose layer located between the dermis and underlying muscle. To reduce partial volume effects and ensure analysis of fat-dominant voxels, voxels within each manually segmented depot with a PDFF < 50% were excluded. The final region of interest (ROI) for each depot thus consisted of voxels that met both the anatomical segmentation criteria and the PDFF > 50% threshold. Mice with fewer than 30 remaining voxels after thresholding were excluded from analysis to prevent bias from small depots with potentially disproportionately high partial volume contamination. Average PDFF, T1, and fatty acid composition metrics (SFA, MUFA, PUFA) were then calculated over this thresholded ROI for each depot. EAT SFA was indexed to the SAT SFA, as SAT is considered a metabolically healthier adipose depot, and our group has previously shown that higher EAT SFA index correlates with proinflammatory macrophage infiltration and cytokine expression in the EAT [25]. Additionally, to assess myocardial lipid, the myocardium was manually segmented and the average PDFF was calculated.

CMR data were excluded from analysis if poor-quality ECG and/or respiratory signals led to severe image artifacts. In total, five ASL measurements, nine DENSE measurements, and eight cine measurements were excluded. Additionally, for FAC and T1 mapping, nine EAT and five myocardial measurements were excluded due to insufficient EAT or myocardial volume in the chosen slice.

### 2.4 Glucose tolerance tests

For GTTs, mice were injected intraperitoneally with sterile glucose (8 g/kg body weight) in deionized water after a 16 h overnight fast. Blood samples were taken from the tail vein before injection to measure the fasting blood glucose, and 10, 30, 60, 90, and 120 min after injection of the glucose solution. The area under the curve (AUC) was calculated by trapezoidal approximation to evaluate glucose tolerance [31]. One HFHS mouse in the SGLT2 inhibitor study was excluded from analysis due to incomplete intraperitoneal delivery of glucose during the experiment.

### 2.5 Vascular reactivity

Mice were euthanized and coronary arterioles were isolated and freed of the surrounding cardiac myocytes. Using an arteriography system (Danish MyoTechnology), the arterioles were cannulated at both ends and pressurized to 40 mm Hg as previously described [32], [33], [34]. Arterioles were pre-constricted with 10 µmol/L phenylephrine. Vessel relaxation measurements are reported as a percent dilation of the initial vessel diameter. Cumulative dose responses to endothelial-independent vasodilators—adenosine and the vascular smooth muscle cell (VSMC)-specific dilator sodium nitroprusside (SNP)—were measured as previously described [32], [33], [34].

### 2.6 Flow cytometry

Methods for flow cytometry are provided in the supplemental information.

### 2.7 Histology

Methods for histology are provided in the supplemental information.

### 2.8 Statistics

Statistical analyses were performed using GraphPad Prism version 10.4.0. (GraphPad Software, Boston, Massachusetts) Normality was evaluated with the Shapiro-Wilk test. A p-value <0.05 was considered statistically significant.

The study was powered to detect MPR differences of 0.4 with a standard deviation of 0.35 between key comparisons across treatment groups and genotypes with 80% power and a significance of 0.05. Using a Student’s t-test for EMPA experiments or a two-way ANOVA with post hoc Šidák’s test for macrophage NOS2 experiments, power analyses show that 10 and 13 mice per group are needed to detect MPR differences of 0.4, respectively. To account for potential exclusions due to unsuccessful CMR, 15 mice per group were used.

For groups with n ≤ 10, non-parametric analyses were performed. Between-group comparisons used the Mann–Whitney U test (two groups) or Kruskal–Wallis test (multiple groups). For vascular reactivity experiments, a Kruskal–Wallis one-way analysis of variance (ANOVA) followed by pairwise Mann–Whitney U tests was used to evaluate differences at each vasodilator dose.

For larger groups where normality was confirmed, two-tailed Student’s t-tests were applied. Two-way ANOVA with Šidák’s post hoc test was used for macrophage NOS2 experiments.

Correlations between GTT AUC and cardiovascular function parameters (global longitudinal strain, PDSR, EF, and MPR) were assessed with Spearman’s rank correlation.

Data are presented as median [interquartile range] for non-parametric analyses and as mean ± standard deviation for parametric analyses.

## 3 Results

### 3.1 SGLT2 inhibitor treatment prevents key features of obesity-induced metabolic heart disease

#### 3.1.1 SGLT2 inhibitor treatment reduces obesity and prevents glucose intolerance

**Fig. 2:**
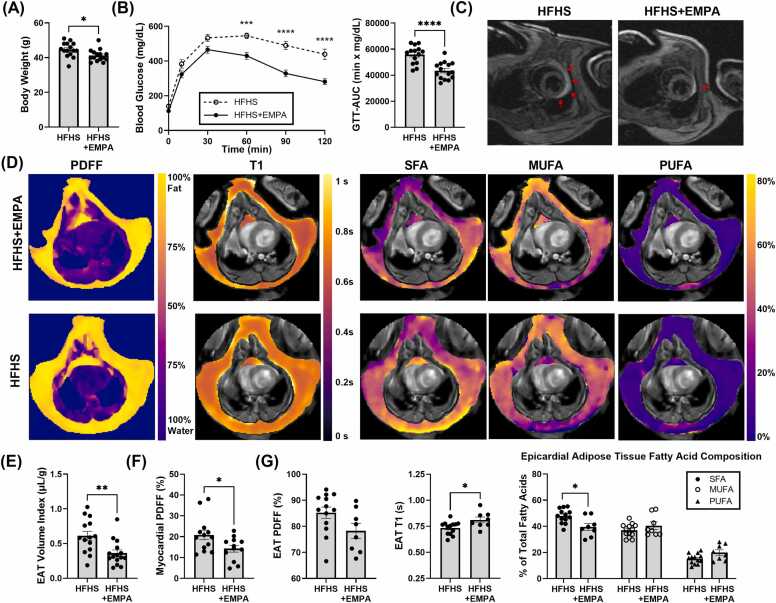
Effect of EMPA treatment on body weight, GTT, myocardial PDFF, and EAT quantity and quality measurements. (A) Body weight in grams for HFHS (n = 15) and HFHS+EMPA (n = 15) mice after 18 weeks of diet. (B) Average glucose tolerance curves and corresponding AUC values for HFHS (n = 14) and HFHS+EMPA (n = 15) mice after 17 weeks of diet. (C) Short-axis black-blood cine images at end-diastole of HFHS and HFHS+EMPA mice after 18 weeks of diet showing greater EAT volume (red arrows) in HFHS mice compared to HFHS+EMPA mice. (D) Example PDFF, T1, SFA, MUFA, and PUFA maps overlayed on SAT and EAT of an HFHS+EMPA mouse (top row) and HFHS mouse (bottom row) after 18 weeks of an HFHS diet. (E) EAT volume index (HFHS: n = 14, HFHS+EMPA: n = 15), (F) myocardial PDFF (HFHS: n = 13, HFHS+EMPA: n = 12), and (G) EAT PDFF, T1, and FAC (SFA/MUFA/PUFA) (HFHS: n = 13, HFHS+EMPA: n = 8) measurements in HFHS and HFHS+EMPA mice after 18 weeks of diet. Data are shown as mean ± SEM and compared using a two-tailed Student’s *t*-test or Mann–Whitney U test as described in Section 2.8.*p<0.05 and **p<0.01 for indicated groups. *EMPA* empagliflozin, *HFHS* high-fat high-sucrose, *GTT* glucose tolerance testing, *CMR* cardiovascular magnetic resonance, *NOS2* inducible nitric oxide synthase, *SCD* standard chow diet, *EAT* epicardial adipose tissue, *PDFF* proton density fat fraction, *SFA* saturated fatty acid fraction, *MUFA* monounsaturated fatty acid fraction, *PUFA* polyunsaturated fatty acid fraction, *SAT* subcutaneous adipose tissue, *FAC* fatty acid composition, *SEM* standard error of the mean

To determine the effect of preventive intervention with the SGLT2 inhibitor, EMPA, on mice fed an HFHSD, markers of cardiometabolic syndrome were assessed, including body weight and glucose tolerance. After 18 weeks on the diet, HFHS+EMPA mice weighed significantly less than HFHS controls (41.20 ± 3.43 g vs 44.67 ± 4.15 g, p<0.05) (Fig. 2A). Glucose tolerance tests and corresponding AUC measurements (Fig. 2B) demonstrated that EMPA significantly reduced the severity of glucose intolerance after 18 weeks of HFHSD (AUC: (43.46 ± 6.55) × 10³ min·mg/dL vs (55.84 ± 6.98) × 10³ min·mg/dL, p<0.0001).

#### 3.1.2 SGLT2 inhibitor treatment prevents excessive EAT accumulation

Representative mid-ventricular short-axis end-diastolic images demonstrate lower EAT volume in HFHS+EMPA mice compared to HFHS mice (Fig. 2C). CMR showed a 40.8% (0.25/0.61) reduction in EAT volume index in HFHS+EMPA mice compared to HFHS controls (0.36 ± 0.18 µL/g vs 0.61 ± 0.25 µL/g, p<0.01) (Fig. 2E).

#### 3.1.3 SGLT2 inhibitor treatment modifies the myocardial PDFF and the FAC and T1 of EAT but not SAT

To assess the effect of SGLT2 inhibitor treatment on preventing myocardial fat accumulation and on biomarkers of proinflammatory EAT, we used CMR to quantify myocardial PDFF and EAT FAC, T1, and PDFF. Example parametric maps overlayed on the EAT and SAT of HFHS+EMPA and HFHS mice are shown in Fig. 2D. Myocardial PDFF values were lower in the HFHS+EMPA mice compared to HFHS controls (14.26 ± 5.70% vs 20.85 ± 8.31%, p<0.05) (Fig. 2F). CMR also demonstrated significant EAT quality changes in HFHS+EMPA mice compared to HFHS controls, including a lower EAT SFA (38.81 [32.83–47.71]% vs 48.06 [43.82–52.65]%, p<0.05) (Fig. 2G) and EAT SFA index (0.94 [0.78–1.00]% vs 1.11 [1.03–1.22]%, p<0.001). There was a trend toward a lower EAT PDFF (78.29 [70.47–86.36]% vs 86.35 [80.18–91.54]%, p=0.075) and EAT T1 was significantly longer (0.799 [0.764–0.859] s vs 0.755 [0.678–0.772] s, p<0.05) in HFHS+EMPA mice compared to controls. For comparison, we quantified the FAC, PDFF, and T1 of SAT from the HFHS+EMPA and HFHS mice. SAT metrics (SFA/MUFA/PUFA, PDFF, and T1) showed no differences between groups (Supplemental Fig. 2).

#### 3.1.4 SGLT2 inhibitor treatment prevents HFHSD-induced coronary microvascular dysfunction

**Fig. 3:**
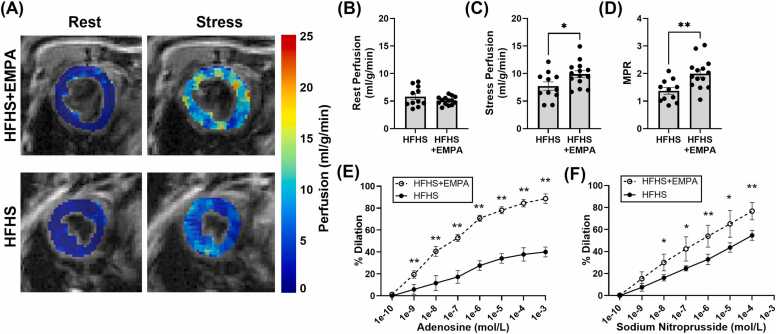
Effect of EMPA treatment on myocardial perfusion, MPR, and coronary arteriole vasoreactivity. (A) Example myocardial perfusion maps acquired at rest and during adenosine-induced stress in a mid-ventricular short-axis slice of an HFHS+EMPA and HFHS mouse. (B) Rest perfusion, (C) stress perfusion, and (D) MPR measurements for HFHS (n=11) and HFHS+EMPA (n=14) mice after 18 weeks of diet. Cumulative arteriolar dose-response curves to (E) adenosine and (F) sodium nitroprusside in HFHS (n=5) and HFHS+EMPA mice (n=5) after 20 weeks of diet. Dose-response curves are shown as mean ± SD. All other data are shown as mean ± SEM and compared using a two-tailed Student’s *t*-test or a Kruskal–Wallis followed by a Mann–Whitney U test as described in Section 2.8.*p<0.05 and **p<0.01 for indicated groups. *MPR* myocardial perfusion reserve, *SD* standard deviation, *RM* repeated measures, *ANOVA* analysis of variance, *EMPA* empagliflozin, *HFHS* high-fat high-sucrose, *GTT* glucose tolerance testing, *CMR* cardiovascular magnetic resonance, *NOS2* inducible nitric oxide synthase, *SCD* standard chow diet, *EAT* epicardial adipose tissue, *PDFF* proton density fat fraction, *SFA* saturated fatty acid fraction, *MUFA* monounsaturated fatty acid fraction, *PUFA* polyunsaturated fatty acid fraction, *SAT* subcutaneous adipose tissue, *FAC* fatty acid composition, *SEM* standard error of the mean

To evaluate coronary microvascular function, we conducted rest and adenosine-induced ASL myocardial perfusion imaging in HFHS+EMPA and HFHS control mice. Example rest and adenosine-stress myocardial perfusion maps are shown in Fig. 3A. Rest MBF was similar between HFHS+EMPA and HFHS mice (5.07 ± 0.75 mL/g/min vs 5.81 ± 1.79 mL/g/min) (Fig. 3B). HFHS+EMPA mice had significantly higher adenosine-induced stress MBF compared to controls (9.91 ± 2.25 mL/g/min vs 7.72 ± 2.51 mL/g/min, p<0.05), resulting in a higher MPR (2.00 ± 0.54 vs 1.37 ± 0.40, p<0.01) (Fig. 3C-D).

To confirm the in vivo CMR perfusion results, we performed ex vivo vasoreactivity testing of isolated coronary arterioles in response to adenosine and SNP. The cumulative dose-response curves to adenosine (p<0.01) and SNP (p<0.05) show significant impairment in dilatory capacity for HFHS mice that is prevented with EMPA treatment (Fig. 3E-F).

To elucidate the relationship between metabolic and cardiovascular parameters, regression analyses were performed between GTT and cardiovascular function, including strain metrics and MPR. Regression analysis demonstrated a significant negative relationship (p<0.05) between glucose tolerance and MPR (Supplemental Fig. 3).

#### 3.1.5 SGLT2 inhibitor treatment prevents HFHSD-induced diastolic dysfunction, and provides lower LV mass and diastolic wall thickness and higher EF

**Fig. 4:**
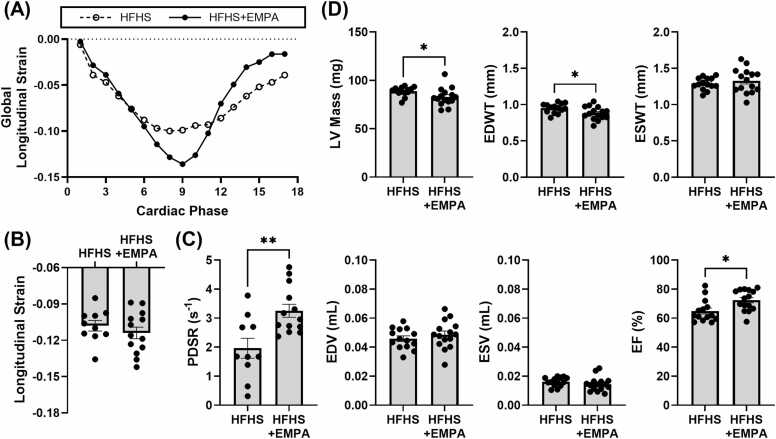
Effect of EMPA treatment on systolic strain, diastolic strain rate, and LV structure and function. (A) Example global longitudinal strain curves in an HFHS and HFHS+EMPA mouse after 18 weeks of diet showing improvements in diastolic function with EMPA. (B) Global longitudinal strain and (C) PDSR measurements in HFHS (n = 10) and HFHS+EMPA (n = 13) mice after 18 weeks of diet. (D) Cine-derived cardiac structure and function parameters including LV mass, EDWT, ESWT, EDV, ESV, and EF in HFHS (n = 14) and HFHS+EMPA (n = 15) mice after 18 weeks of diet. Data are shown as mean ± SEM and compared using a two-tailed Student’s *t*-test or Mann–Whitney U test as described in Section 2.8.*p<0.05 and **p<0.01 for indicated groups. *PDSR* peak diastolic strain rate, *LV* left ventricular, *EDWT* end-diastolic wall thickness, *ESWT* end-systolic wall thickness, *EDV* end-diastolic volume, *ESV* end-systolic volume, *EF* ejection fraction, *EMPA* empagliflozin, *HFHS* high-fat high-sucrose, *GTT* glucose tolerance testing, *CMR* cardiovascular magnetic resonance, *NOS2* inducible nitric oxide synthase, *SCD* standard chow diet, *EAT* epicardial adipose tissue, *PDFF* proton density fat fraction, *SFA* saturated fatty acid fraction, *MUFA* monounsaturated fatty acid fraction, *PUFA* polyunsaturated fatty acid fraction, *SAT* subcutaneous adipose tissue, *FAC* fatty acid composition, *SEM* standard error of the mean

We evaluated the effect of SGLT2 inhibitor treatment on systolic and diastolic cardiac function using DENSE CMR. There was a trend toward improved peak global longitudinal end-systolic strain in the HFHS+EMPA mice compared to controls, though it was not significant (Fig. 4B, representative curves Fig. 4A). The diastolic strain rate was higher in the HFHS+EMPA mice compared to controls (2.96 [2.61–3.99] s^-1^ vs 1.68 [1.21–2.80] s^-1^, p<0.01) (Fig. 4C), indicating better diastolic function.

Cine imaging showed that HFHS+EMPA and HFHS mice had similar EDV, ESV, and ESWT (Fig. 4D). LV mass was lower in HFHS+EMPA mice compared to HFHS mice (82.84 ± 9.26 mg vs 88.64 ± 4.69 mg, p<0.05) (Fig. 4D). EDWT was lower in HFHS+EMPA mice compared to controls (0.88 ± 0.09 mm vs 0.95 ± 0.06 mm, p<0.05) (Fig. 4D). HFHS+EMPA mice had a higher EF compared to HFHS mice (72.42 ± 7.17% vs 64.90 ± 7.73%, p<0.05), although both groups maintained a preserved EF (>50%) (Fig. 4D).

#### 3.1.6 SGLT2 inhibitor treatment prevents adipocyte hypertrophy

**Fig. 5:**
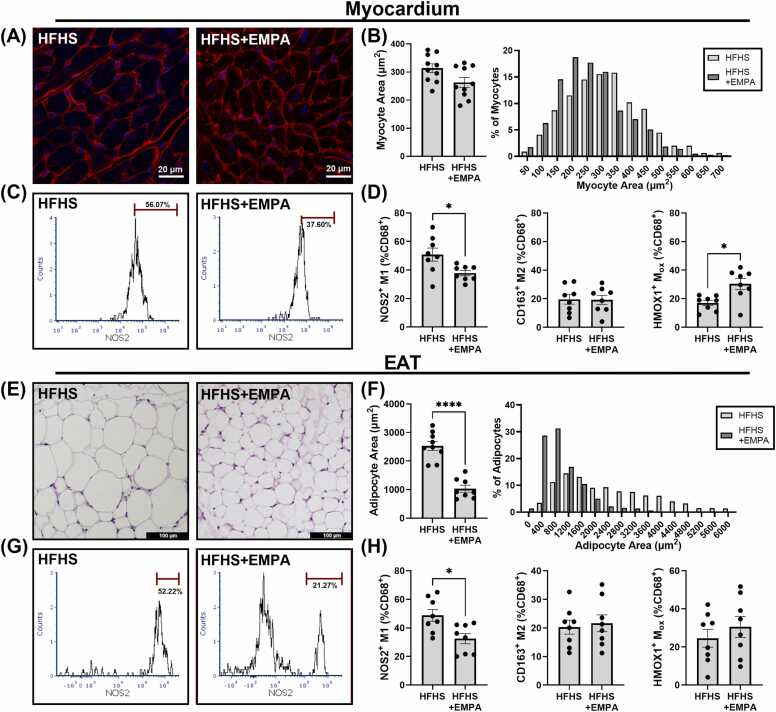
Effect of EMPA treatment on cardiomyocyte and EAT adipocyte hypertrophy and macrophage polarization. (A) WGA (red) staining of cardiac myocytes and (B) quantification and distribution of myocyte size in HFHS (n = 10) and HFHS+EMPA (n = 10) mice after 20 weeks of diet. (C) Flow cytometry histograms depicting higher levels of NOS2^+^ macrophages isolated from the hearts of mice fed an HFHS or HFHS+EMPA for 20 weeks. (D) Flow cytometry analysis of NOS2^+^ cells, CD163^+^ cells, and HMOX1^+^ cells as a percentage of CD68^+^ cells isolated from the hearts of mice fed an HFHS (n = 8) or HFHS+EMPA (n = 8) for 20 weeks. (E) H&E staining of EAT and (F) quantification and distribution of adipocyte size in HFHS (n = 9) and HFHS+EMPA mice (n = 8) after 20 weeks of diet. (G) Flow cytometry histograms depicting higher levels of NOS2^+^ macrophages isolated from the EAT of mice fed an HFHS or HFHS+EMPA for 20 weeks. (H) Flow cytometry analysis of NOS2^+^ cells, CD163^+^ cells, and HMOX1^+^ cells as a percentage of CD68^+^ cells isolated from the EAT of mice fed an HFHS (n = 8) or HFHS+EMPA (n = 8) for 20 weeks. Data are shown as mean ± SEM and compared using a Mann–Whitney U test. *p<0.05 and ****p<0.0001 for indicated groups. *WGA* wheat germ agglutinin, *H&E* hematoxylin and eosin, *EMPA* empagliflozin, *HFHS* high-fat high-sucrose, *GTT* glucose tolerance testing, *CMR* cardiovascular magnetic resonance, *NOS2* inducible nitric oxide synthase, *SCD* standard chow diet, *EAT* epicardial adipose tissue, *PDFF* proton density fat fraction, *SFA* saturated fatty acid fraction, *MUFA* monounsaturated fatty acid fraction, *PUFA* polyunsaturated fatty acid fraction, *SAT* subcutaneous adipose tissue, *FAC* fatty acid composition, *SEM* standard error of the mean

To evaluate the impact of SGLT2 inhibitor treatment on cardiomyocyte size and adipocyte hypertrophy as indicators of cardiac hypertrophy and proinflammatory EAT quality in obesity-related MHD, respectively, we analyzed WGA-stained sections of heart tissue and H&E-stained sections of EAT from HFHS+EMPA and HFHS mice. Representative histological sections of myocardial tissue (Fig. 5A) showed that HFHS+EMPA mice exhibited a trend toward reduced cardiomyocyte hypertrophy compared to HFHS controls, as indicated by smaller mean cardiomyocyte cross-sectional area (252.3 [214.8–322.0] µm^2^ vs 316.2 [270.8–364.6] µm^2^, p = 0.06) (Fig. 5B). Representative histological sections of EAT (Fig. 5E) showed that HFHS+EMPA mice exhibited reduced adipocyte hypertrophy compared to HFHS controls, as indicated by smaller mean adipocyte area (910.8 [723.7–1336.7] µm^2^ vs 2484.8 [2095.6–2945.5] µm^2^, p<0.001) (Fig. 5F).

#### 3.1.7 SGLT2 inhibitor treatment prevents EAT macrophage infiltration, and shifts heart and EAT macrophage polarization toward a less proinflammatory state

To investigate the relationship between CMR findings, SGLT2 inhibitor treatment, and shifts in macrophage infiltration and polarization, we performed histopathological analysis of F4/80^+^ macrophages, a pan macrophage marker, in the EAT. Histopathological analysis revealed a significantly lower number of macrophages (F4/80^+^ positive puncta) in the EAT of HFHS+EMPA mice compared to HFHS mice (Supplemental Fig. 4). To quantify inflammatory infiltrates, flow cytometry of select macrophage populations (CD68^+^) in the hearts and EAT of HFHS+EMPA and HFHS mice was performed. Analysis revealed a significantly lower percentage of NOS2^+^ (M1 – proinflammatory) macrophages in HFHS+EMPA mice in both the heart (38.42 [33.91–41.89]% vs 50.44 [42.08–61.04]%, p<0.05) (Fig. 5C-D) and EAT (34.74 [21.38–42.098]% vs 46.36 [38.08–61.30]%, p<0.05) (Fig. 5G-H) compared to HFHS controls. Meanwhile, no significant differences were observed in CD163^+^ (M2 – proresolving/anti-inflammatory) macrophage levels in the hearts (Fig. 5D) or EAT (Fig. 5H) between the two groups. Interestingly, in the hearts of HFHS+EMPA mice, we identified a higher population of HMOX1^+^ (Mox) macrophage levels compared to HFHS mice (32.70 [24.70–37.93]% vs 18.82 [11.54–21.60]%, p<0.05) (Fig. 5D).

### 3.2 Genetic suppression of macrophage NOS2 preserves adenosine MPR but does not prevent diastolic dysfunction

#### 3.2.1 Genetic suppression of macrophage NOS2 does not affect HFHSD-induced weight gain or glucose intolerance

**Fig. 6:**
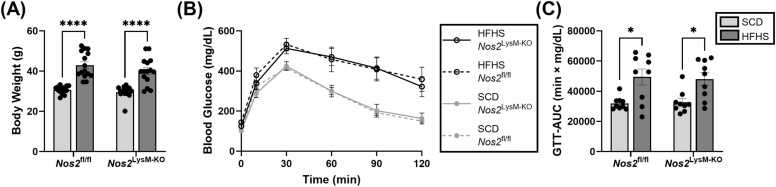
Effect of macrophage NOS2 on body weight and GTT. (A) Body weight in grams for *Nos2*^fl/fl^ and *Nos2*^LysM-KO^ mice fed an HFHS diet or SCD for 18 weeks (n=15/group). (B) Average glucose tolerance curves and (C) corresponding AUC values for *Nos2*^fl/fl^ and *Nos2*^LysM-KO^ mice fed an HFHS diet or SCD for 17 weeks (n=9/group). Data are shown as mean ± SEM and compared using a two-way ANOVA with Šidák’s multiple comparisons test or Kruskal–Wallis test with Dunn’s multiple comparisons test as described in Section 2.8. *p<0.05 and ****p<0.0001 for either HFHS group vs SCD group. *EMPA* empagliflozin, *HFHS* high-fat high-sucrose, *GTT* glucose tolerance testing, *CMR* cardiovascular magnetic resonance, *NOS2* inducible nitric oxide synthase, *SCD* standard chow diet, *EAT* epicardial adipose tissue, *PDFF* proton density fat fraction, *SFA* saturated fatty acid fraction, *MUFA* monounsaturated fatty acid fraction, *PUFA* polyunsaturated fatty acid fraction, *SAT* subcutaneous adipose tissue, *FAC* fatty acid composition, *SEM* standard error of the mean

Building on the findings that EMPA administration prevents EAT inflammation, NOS2^+^ M1 macrophages in the EAT and myocardium, and impairments in adenosine MPR and diastolic function, combined with previous research showing the key roles of NOS2 in diastolic dysfunction and CMD [2], [3], we aimed to investigate whether NOS2-expressing macrophages specifically contribute to impaired adenosine MPR and diastolic dysfunction. In these studies, male *Nos2*^LysM-KO^ and *Nos2*^fl/fl^ control mice were fed either an HFHSD or an SCD for 18 weeks. After 18 weeks, both HFHSD groups showed similar weight gain and glucose intolerance, with significantly greater weight gain (*Nos2*^LysM-KO^ HFHSD vs *Nos2*^LysM-KO^ SCD: 40.05 ± 6.60 g vs 29.57 ± 2.99 g, p = <0.0001; *Nos2*^fl/fl^ HFHSD vs *Nos2*^fl/fl^ SCD: 42.95 ± 6.42 g vs 30.71 ± 1.88 g, p = <0.0001) and elevated glucose intolerance (*Nos2*^LysM-KO^ HFHSD AUC vs *Nos2*^LysM-KO^ SCD AUC: (51.54 [35.30–60.81]) × 10^3^ min·mg/dL vs (31.29 [27.74–35.33]) × 10^3^ min·mg/dL, p<0.05; *Nos2*^fl/fl^ HFHSD AUC vs *Nos2*^fl/fl^ SCD AUC: (52.86 [32.93–62.87]) × 10^3^ min·mg/dL vs (30.14 [28.60–33.74]) × 10^3^ min·mg/dL, p<0.05) compared to their respective SCD groups (Fig. 6A-C).

#### 3.2.2 Genetic suppression of macrophage NOS2 prevents HFHSD-induced coronary microvascular dysfunction

**Fig. 7:**
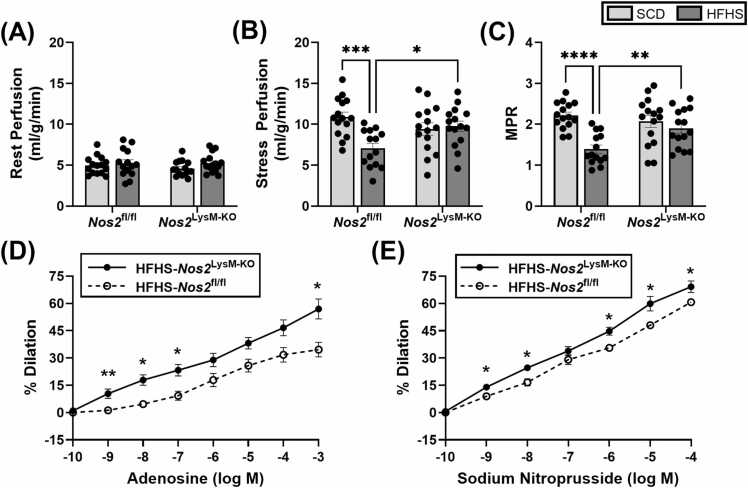
Effect of macrophage NOS2 on myocardial perfusion, MPR, and coronary arteriole vasoreactivity. (A) Rest perfusion, (B) adenosine-induced stress perfusion, and (C) MPR for *Nos2*^fl/fl^ (SCD: n=15, HFHS: n=14) and *Nos2*^LysM-KO^ (n=15/group) mice fed an HFHS diet or SCD for 18 weeks. Cumulative arteriolar dose-response curves to (D) adenosine and (E) sodium nitroprusside in *Nos2*^fl/fl^ and *Nos2*^LysM-KO^ mice (n = 5/group) after 20 weeks of HFHS diet. Data are shown as mean ± SEM. Dose-response curves are compared using a Kruskal–Wallis test followed by a Mann–Whitney U test. All other data is compared using an ordinary two-way ANOVA with Šidák’s multiple comparisons test. *p<0.05, **p<0.01, ***p<0.001, and ****p<0.0001 for indicated groups. *EMPA* empagliflozin, *HFHS* high-fat high-sucrose, *GTT* glucose tolerance testing, *CMR* cardiovascular magnetic resonance, *NOS2* inducible nitric oxide synthase, *SCD* standard chow diet, *EAT* epicardial adipose tissue, *PDFF* proton density fat fraction, *SFA* saturated fatty acid fraction, *MUFA* monounsaturated fatty acid fraction, *PUFA* polyunsaturated fatty acid fraction, *SAT* subcutaneous adipose tissue, *FAC* fatty acid composition, *SEM* standard error of the mean

Rest myocardial blood flow was similar across all groups (Fig. 7A). However, after 18 weeks of an HFHSD, while stress perfusion was significantly reduced in *Nos2*^fl/fl^ mice compared to SCD controls (7.07 ± 2.25 mL/g/min vs 10.89 ± 2.36 mL/g/min, p<0.001), it was preserved in the *Nos2*^LysM-KO^ mice compared to SCD controls mice (9.77 ± 2.43 mL/g/min vs 9.43 ± 2.92 mL/g/min) (Fig. 7B). Accordingly, MPR was reduced in *Nos2*^fl/fl^ mice on an HFHSD compared to SCD controls (1.39 ± 0.38 vs 2.21 ± 0.32, p<0.0001), but maintained in *Nos2*^LysM-KO^ mice compared to SCD controls (1.90 ± 0.47 vs 2.07 ± 0.53) (Fig. 7C). Stress perfusion (9.77 ± 2.43 mL/g/min vs 7.07 ± 2.25 mL/g/min, p<0.05) and MPR (1.90 ± 0.47 vs 1.39 ± 0.38, p<0.01) were also greater in *Nos2*^LysM-KO^ mice fed an HFHSD compared to *Nos2*^fl/fl^ mice fed an HFHSD.

Ex vivo vasoreactivity tests showed preservation of coronary arteriolar dilation in *Nos2*^LysM-KO^ mice compared to *Nos2*^fl/fl^ control mice on a HFHSD, as reflected in cumulative dose-response curves to adenosine (p<0.05) and SNP (p<0.05) (Fig. 7D-E).

#### 3.2.3 Genetic suppression of macrophage NOS2 does not prevent HFHSD-induced diastolic dysfunction

Trends of reduced peak global end-systolic strain were seen in HFHSD groups compared to SCD groups (Supplemental Fig. 5A), but peak global end-systolic strain was similar between HFHSD-*Nos2*^LysM-KO^ and HFHSD-*Nos2*^fl/fl^ mice. After 18 weeks of HFHSD, there was a trend toward a decrease in PDSR in HFHSD mice compared to SCD controls (Supplemental Fig. 5B). No differences in PDSR were observed between HFHSD-*Nos2*^LysM-KO^ and HFHSD-*Nos2*^fl/fl^ mice. These results suggest that myeloid-specific NOS2 does not significantly impact diastolic or systolic dysfunction under HFHSD conditions. Trends of small increases in LV mass, EDWT, and ESWT were seen in both HFHSD groups compared to SCD groups, however, no significant differences in cine-derived metrics of cardiac structure or function were observed between groups after 18 weeks on either diet (Supplemental Fig. 5C).

## 4 Discussion

The major findings of these studies utilizing multiparametric CMR in an HFHSD-induced mouse model of obesity-induced MHD are that SGLT2 inhibition initiated concurrently with the start of an 18-week HFHSD prevented impairment of MPR and diastolic dysfunction, and also prevented the accumulation of EAT volume, CMR proinflammatory EAT biomarkers, and NOS2^+^ macrophage M1 polarization. Furthermore, knockout of macrophage NOS2 led to preserved adenosine MPR but not to preserved diastolic function after 18 weeks of an HFHSD.

These results are consistent with a disease mechanism model where obesity drives the accumulation of proinflammatory EAT and myocardial lipid, which promote myocardial and vascular dysfunction. Saturated fatty acid overload in hypertrophic adipocytes leads to TLR4 pathway activation, causing proinflammatory cytokine secretion (e.g., MCP-1, IL-6, TNF-α) by adipocytes and macrophage recruitment and M1 polarization in the EAT [6]. Inflammatory cytokines are secreted into the shared microcirculation and transmitted to the myocardium via vasocrine and paracrine signaling [35], [36]. This leads to an increase in NOS2^+^ M1-polarized macrophages in the myocardium where they exacerbate oxidative and nitrosative stress. These processes collectively contribute to myocardial and vascular dysfunction, linking obesity and EAT inflammation to the pathophysiology of metabolic heart disease.

Our results contribute to a deeper understanding of the critical roles of NOS2 in obesity-induced MHD. Prior work from our group showed that global *Nos2* deletion completely preserved myocardial perfusion reserve and diastolic function by CMR, which are otherwise impaired by an HFHSD [3]. Additionally, treatment with 1400 W, an in vivo inhibitor of NOS2, applied after the establishment of impaired MPR and diastolic function, partially reversed CMD and prevented worsening diastolic dysfunction caused by an HFHSD [3]. These findings were consistent with those of Schiattarella et al., where pharmacological or genetic suppression of NOS2 ameliorated LV diastolic dysfunction and exercise intolerance in a high-fat diet + L-NAME mouse model of HFpEF [2]. In the present study, macrophage-specific NOS2 knockout restored MPR but failed to alleviate diastolic dysfunction caused by an HFHSD. These findings show the role of macrophage NOS2 in obesity-driven coronary microvascular dysfunction, while suggesting that other NOS2 sources may play a role in diastolic dysfunction.

This study shows the potential of new CMR adipose tissue imaging methods, recently developed for mice [14], [25] and humans [24], that quantify biomarkers of proinflammatory EAT and myocardial lipid, including EAT FAC (SFA, MUFA, PUFA, and these values indexed to their SAT counterparts), PDFF, and T1, as well as myocardial PDFF. Our findings suggest that high SFA, low unsaturated fatty acids, and short T1 values reflect a proinflammatory state in EAT. SGLT2 inhibitor treatment with EMPA prevented changes in EAT SFA and T1, while leaving the FAC and T1 of SAT unchanged, underscoring the specific effects of EMPA on VAT, particularly EAT. The FAC differences are consistent with known effects of SGLT2 inhibitors on lipid metabolism, including reduced lipogenesis and increased lipolysis [37]. The T1 changes, which are consistent with findings of higher VAT T1 in healthy individuals compared to those with obesity [38] and after bariatric surgery [39], may reflect reduced reactive oxygen species, altered FAC, adipose tissue browning, or morphological changes such as smaller adipocytes and fewer crown-like structures. These findings align with our observation of less macrophage infiltration and smaller adipocyte size due to EMPA treatment. Furthermore, SGLT2 inhibitor treatment with EMPA significantly prevented increased myocardial PDFF, indicating less myocardial fat accumulation. While previous studies consistently show that SGLT2 inhibitors reduce EAT volume [40], [41], [42] and adipose tissue inflammation [12], [43], [44], [45], our results provide further insight into EAT-specific FAC changes, including reduced SFA and prolonged T1 relaxation times detectable through noninvasive CMR. Further investigation is warranted to establish the potential clinical utility of CMR-derived biomarkers, such as EAT FAC and T1, in clinical studies of obesity-induced MHD.

Our study adds to the growing evidence that SGLT2 inhibitors promote anti-inflammatory macrophage polarization. To our knowledge, the present data are the first to show prevention of EAT macrophage M1 polarization with SGLT2 inhibitor treatment. We observed a similar effect in the heart after preventive SGLT2 inhibitor treatment, with fewer M1 macrophages and more Mox macrophages. These findings align with prior research showing that SGLT2 inhibition reduces M1 macrophages in the epididymal adipose tissue [12], and increases M2 macrophage infiltration in infarcted rat hearts [46]. Interestingly, the increase in myocardial antioxidant Mox macrophages with EMPA treatment is a novel finding that may reflect enhanced antioxidant capacity, helping to counteract the elevated oxidative stress induced by an HFHSD [47].

These experiments firmly established that SGLT2 inhibitor treatment prevents an impaired coronary microvascular response to adenosine receptor agonism. While previous studies have presented results related to this effect, our results unambiguously show that EMPA prevents an impaired response of the coronary microvessels to adenosine receptor agonism without complication from obstructive coronary artery disease (CAD) [48]. Arterial spin labeling, the technique used to quantify perfusion in this study, has been carefully validated previously in the rat heart using microspheres as a reference [49]. In mice, where microsphere analysis is technically challenging, the same ASL methods have been validated against first-pass gadolinium-enhanced MRI, showing good agreement for quantification of myocardial perfusion in mice over a large range of perfusion values [20]. In a study of patients with stable CAD, Leccisotti et al. [48] reported improved myocardial flow reserve with SGLT2 inhibitor treatment using adenosine-induced hyperemia and PET/CT imaging. Adingupu et al. [16] demonstrated that SGLT2 inhibitor treatment improved coronary flow velocity reserve based on isoflurane-induced hyperemia and ultrasound imaging in an ob/ob^-/-^ mouse model of obesity. Unlike these studies, we used adenosine—a clinically relevant vasodilator—in a model of coronary microvascular disease without atherosclerosis. This allowed us to directly measure the improved coronary microvascular response to adenosine receptor agonism, as reflected in the increased MPR. This finding was supported by ex vivo coronary microvascular reactivity experiments, which are the first to directly quantify enhanced adenosine-induced dilation of small coronary vessels with SGLT2 inhibitor treatment.

We observed prevention of impaired diastolic function with EMPA treatment. Quantitative assessments of myocardial strain-time curves showed a significant increase in PDSR with EMPA compared to untreated mice. These findings are in line with prior preclinical and clinical studies investigating SGLT2 inhibitor treatment and cardiac diastolic function. Habibi et al. [10] found that EMPA improved diastolic function using Doppler echocardiography in female diabetic mice, and Verma et al. [11] identified a similar improvement in tissue Doppler-derived diastolic function in diabetic patients treated with EMPA. Additionally, a randomized, controlled trial in patients with diabetes found that treatment with the SGLT2 inhibitor, dapagliflozin, was associated with a significant improvement in LV diastolic dysfunction assessed with diastolic stress echocardiography compared with placebo [50]. Given that diastolic dysfunction is central to HFpEF, and diastolic function metrics are independent predictors of heart failure incidence [51], [52], our findings highlight the potential of SGLT2 inhibitor treatment to improve outcomes in individuals with MHD at risk for HFpEF.

SGLT2 inhibitors were initially indicated for the treatment of diabetes. Importantly, SGLT2 inhibitors are now considered standard of care for patients with systolic or diastolic heart failure, even in the absence of diabetes [8], [53]. The exact mechanisms through which SGLT2 inhibitors mediate beneficial cardiac effects are likely multifactorial and incompletely understood. Clinically, identifying abnormal glucose metabolism, as indicated by elevated hemoglobin A1c or random blood glucose levels, serves as a clinical marker to initiate early therapy. In addition to using SGLT2 inhibitors to treat biomarkers of diabetes, the present study elucidates how this strategy not only treats diabetes but also simultaneously promotes cardiovascular protection against future impairments to coronary microvascular function, diastolic function, myocardial lipid, and epicardial adipose tissue volume and composition when initiated early.

## 5 Limitations

Despite its contributions, this study has limitations. The specific molecular mechanisms by which SGLT2 inhibition with EMPA improves EAT quality, myocardial perfusion, and diastolic function were not the focus of this study, emphasizing the need to further investigate the interplay between adipose tissue quantity and composition, macrophage-driven inflammation, and cardiovascular dysfunction. This study did not assess circulating biomarkers such as plasma fatty acids or inflammatory cytokines. Future studies incorporating these surrogate markers could help improve clinical translatability of these findings. While this study provides insights into the cardioprotective effects of SGLT2 inhibitor treatment, it does not determine the suitability of SGLT2 inhibitors for patients with obesity-induced MHD. Our study was designed to test the preventive effects of SGLT2 inhibitor treatment given at the initiation of an HFHSD. Evaluating the suitability of SGLT2 inhibition as a therapeutic intervention after disease onset is an important direction for future research.

Studies were performed in male mice, thus potential sex differences were not investigated. While male mice develop a robust cardiometabolic phenotype in response to HFHS feeding, female mice, particularly in the premenopausal phase, exhibit a milder cardiac phenotype [54]. However, recent studies demonstrate that post-menopausal female mice develop more severe metabolic and cardiac dysfunction, making them a more relevant model for studying obesity-induced MHD in females [55], [56]. These findings underscore the importance of age and hormonal status in disease susceptibility and support the need for future studies evaluating SGLT2 inhibition in post-menopausal female mouse models that better reflect the clinical complexity of this condition.

Myocardial T1 and T2 mapping were not included in this study due to limitations in scan time and the lack of advanced methods for T2 mapping on our preclinical system. However, these measures may provide additional insight into myocardial tissue characteristics, including diffuse fibrosis and edema. Future studies incorporating these markers may help further clarify the impact of SGLT2 inhibition on myocardial inflammation and remodeling. Because images were manually segmented by a non-blinded operator, the potential for bias cannot be excluded, although prior studies have demonstrated good reproducibility of these methods in small animal CMR [20], [57].

This study used a LysM-Cre mouse strain to selectively knockout NOS2, and while this strain is an effective tool to target macrophages, other myeloid cells (e.g., neutrophils, granulocytes, dendritic cells) may also be affected, leaving open the possibility that non-macrophage myeloid cells contribute to NOS2-driven microvascular dysfunction. However, macrophages remain the primary contributors to inflammation in both adipose tissue and the myocardium [58], [59]. The roles of non-myeloid NOS2 sources, such as vascular smooth muscle cells and cardiomyocytes, in diastolic dysfunction were not explored. Future studies should address other cell types to provide a more comprehensive understanding of NOS2 in cardiovascular disease. Additionally, this study did not directly evaluate the effects of EMPA in *Nos2*^LysM-KO^ mice. Future studies administering SGLT2 inhibitors to NOS2-knockout mice may clarify which SGLT2 effects are independent of NOS2 macrophages.

Lastly, blood pressure was not measured in this study, limiting assessment of its role in the findings. Given that EMPA can lower blood pressure [60], future studies should measure blood pressure to clarify the extent to which it contributes to the cardiovascular benefits of SGLT2 inhibition.

## 6 Conclusion

Multiparametric CMR and other experimental methods were used to show the efficacy of SGLT2 inhibitor treatment in preventing proinflammatory EAT changes detectable on CMR, proinflammatory macrophage polarization, myocardial lipid accumulation, and impairment of MPR and diastolic dysfunction in an HFHSD mouse model. Further experiments using a myeloid-specific NOS2 knockout model showed a key role of macrophage NOS2 in coronary microvascular dysfunction. In these contexts, novel CMR biomarkers such as EAT FAC and T1 and myocardial PDFF show utility for detecting key and modifiable adipose-related features of obesity-induced MHD.

## Funding

This work was supported by the National Institutes of Health (NIH) National Heart, Lung, and Blood Institute (R01 HL162872), Bethesda, Maryland; National Institutes of General Medical Sciences Medical Scientist Training Grant Program T32GM007267, Bethesda, Maryland; American Heart Association Pre-Doctoral Fellowship 23PRE1011280, Dallas, Texas; NIH Instrumentation Grant Program S10OD025024, Bethesda, Maryland; and NIH National Cancer Institute P30-CA044579, Bethesda, Maryland.

## Author contributions

J.E.B., C.M.P, F.H.E., and M.J.W. were involved in conceptualization and design of the study. J.E.B. and R.J.R. performed CMR experiments. J.E.B. performed image analysis, statistical analysis, and data visualization. J.E.B. and L.A.B. performed GTT experiments. J.E.B., T.P.S., and J.T.E were involved in the development of pulse sequences, image reconstruction, and image analysis. C.M.P., M.J.W., and L.A.B. performed histology and flow cytometry experiments and analysis. E.H.M. and B.E.I performed vascular reactivity experiments. A.M. provided mice for generation of *Nos2*^LysM-KO^ line. J.E.B., F.H.E., C.M.P., M.J.W. were involved in writing of the original manuscript. All authors critically revised the paper and have read and approved the final manuscript.

## Declaration of generative AI and AI-assisted technologies in the writing process

During the preparation of this work the authors used ChatGPT in order to improve readability and clarity of the manuscript text. After using this tool, the authors reviewed and edited the content as needed and take full responsibility for the content of the publication.

## Declaration of competing interests

The authors declare the following financial interests/personal relationships which may be considered as potential competing interests: Frederick Epstein reports financial support was provided by National Heart Lung and Blood Institute. Julia Bresticker reports financial support was provided by American Heart Association Inc. Christopher Kramer reports a relationship with Eli Lilly and Company that includes: consulting or advisory and funding grants. Amit Patel is an associate editor of JCMR. Other authors declare that they have no known competing financial interests or personal relationships that could have appeared to influence the work reported in this paper.
