# Supplementary material for: Multiparametric cardiac magnetic resonance identifies macrophage nitric oxide synthase 2-mediated benefits of preventive sodium-glucose cotransporter 2 inhibition in a mouse model of metabolic heart disease
Source: J Cardiovasc Magn Reson. 2025 Oct 10;27(2):101972. doi: 10.1016/j.jocmr.2025.101972 (PMC12719530; doi:10.1016/j.jocmr.2025.101972)
Supplement: Supplementary file 1 — Supplementary material [file mmc1.docx]

**Supplemental Information**

**Supplemental Methods**

**Flow cytometry**

Primary infiltrating leukocytes were isolated from cardiac tissue by non-Langendorff perfusion as previously described[1]. Briefly, hearts were sequentially perfused with EDTA buffer, perfusion buffer, and digestion buffer containing collagenase until hearts appeared soft. Hearts were then mechanically dissociated with scissors and pipetted up and down. Cells were washed through a 100 μm cell strainer to create a single cell suspension. Myocytes were isolated by centrifugation at 120 × g for 5 minutes. Supernatants (containing non-myocyte cells) were transferred to clean tubes and pelleted by centrifugation at 300 × g for 5 minutes, then resuspended in 1 mL of FACS buffer (PBS, 2 mM EDTA, and 1% BSA). Splenocytes were isolated by grinding through a 100 μm cell strainer and washing with PBS containing 2 mM EDTA, then treated with ACK Lysis buffer for 5 minutes. EAT was digested with collagenase 2 containing DMEM with gentle shaking at 37°C for 30 minutes. Samples were centrifuged at 700 × g for 5 minutes and stromal vascular fractions (SVF) were collected. All samples were then counted and resuspended at 100 μL per 100,000 cells in PBS. Cells were stained with LIVE/DEAD Yellow (1:1000) (Thermo Scientific L34967) for 30 minutes on ice. Fc-receptors were then blocked for 15 minutes using FcBlock (BioRad – BUF041A). Cells were pelleted by centrifugation (300 × g, 5 minutes) and resuspended in FACS buffer (100 μL per 100,000 cells) and stained with fluorophore-conjugated antibodies (1:100) for 1 hour on ice (Supplemental Table 1). Cells were washed in FACS (×3) after staining. Intracellular staining was performed after fixation and permeabilization using Fix and Perm Kit (BDBiosciences) per manufacturer instructions. Cells were subsequently stained for intracellular markers using fluorophore-conjugated antibodies (1:100) for 1 hour on ice. Cells were washed in wash buffer (BDBiosciences) (×3) after staining. Flow cytometry collection and deconvolution was performed on an Aurora Borealis 5 laser Spectral Flow Cytometer. Automatic deconvolution was performed using single stains generated from splenocytes after setting gating on unstained control cells. FMO controls were collected and used to determine gating strategies. Gating was applied identically across all samples. Gating and post-hoc analysis was performed with FCS-Express 7.18. Representative gating for flow cytometry experiments is presented in Supplemental Figure 1.

**Histology**

Hearts and EAT were collected after CO_2_ induced death. Hearts were fixed for 4 hours in NFB prior to being moved to 70% ethanol. Hearts were not arrested prior to harvest. Hearts were paraffin embedded and cryosectioned to 7 μm thickness and mounted. EAT was fixed in 4% PFA in PBS for 7-10 days at 4°C. EAT was paraffin embedded and cryosectioned at 5 μm thickness and mounted. Slides were deparaffinized. Briefly, sections were submerged in xylene (3 minutes), 1:1 xylene:ethanol (3 minutes), 100% ethanol (2×3 minutes), 95% ethanol (3 minutes), 70% ethanol (3 minutes), and 50% ethanol (3 minutes). Antigen retrieval was performed using citrate-based solution (Vector Laboratories H-330) where slides were submerged in antigen retrieval solution and heated to boiling for 20 minutes, slides were then cooled for 1 hour at room temperature. Tissue sections were then blocked for 1 hour in antibody blocking buffer (FGS, donkey serum) at room temperature. Antibody blocking buffer was removed and replaced with antibody blocking buffer containing primary antibody overnight at 4°C. Sections were then washed (PBS+FGS+Tween for 5 minutes, 2× PBS 5 minutes) and incubated in antibody blocking buffer containing secondary antibody (1:100) for 1 hour at room temperature protected from light. Sections were washed (3× PBS) and counterstained with DAPI (Thermo Fisher Scientific D3571) before mounting. Sections were imaged on an Olympus Fluoview 1000 and are representative images of composite z-stacks. Analysis (thresholding and manual counting) was performed in ImageJ.

| Target | Fluorophore | Producer | Catalog Number | Lot Number |
| --- | --- | --- | --- | --- |
| CD45 - Flow | eFluor 605 | Invitrogen | 69-0451-82 | 2892626 |
| CD68 - Flow | PE | Invitrogen | 12-0681-82 | 2925560 |
| CD163 - Flow | FITC | Invitrogen | 11-1631-82 | 2653045 |
| iNOS (NOS2) - Flow | PerCP-eFluor 710 | Invitrogen | 46-5920-82 | 2626673 |
| HMOX1 - Flow | CoraLite 594 | Proteintech | CL594-66743 | 21017731 |
| Live/Dead Yellow - Flow |  | Invitrogen | L34967A | 2775959 |
| Wheat Germ Agglutinin - IHC | Texas Red | Invitrogen | W21405 |  |
| F4/80 - IHC |  | Invitrogen | MF48000 | 2641995 |

**Supplemental Table 1.** List of antibodies used for flow cytometry and tissue staining.

**
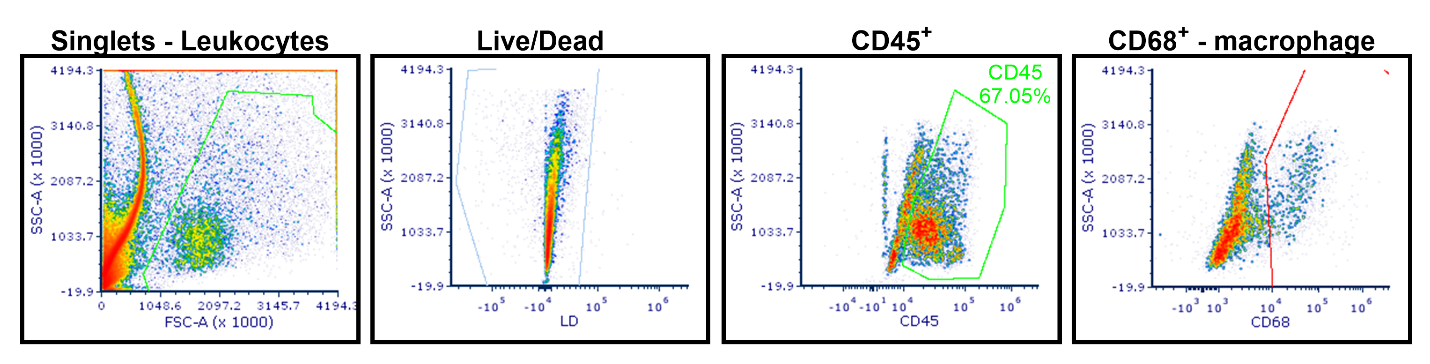
**

**Supplemental Figure 1.** Gating scheme and representative gates for the identification of CD68^+^ macrophage cells from primary immune cells isolated from myocardium and epicardial adipose tissue. Cells were gated for live singlet leukocytes and then CD45^+^ and CD68^+^ cells were identified.


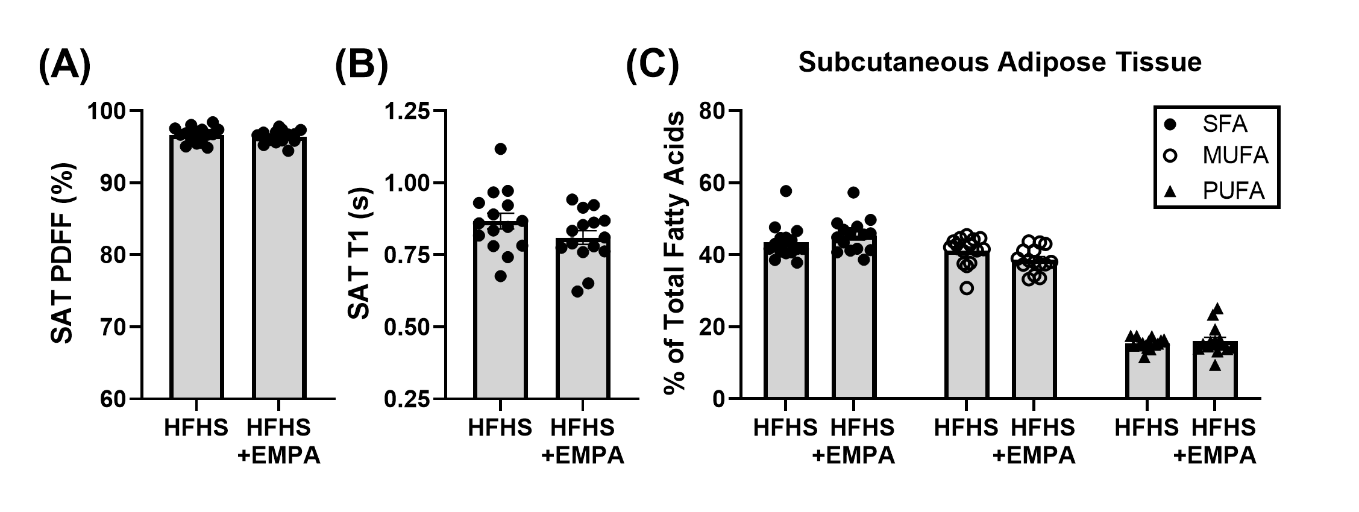


**Supplemental Figure 2. Effect of EMPA treatment on SAT quality.** SAT (A) PDFF, (B) T1, and (C) fatty acid composition (SFA/MUFA/PUFA) in mice (n=15/group) fed an HFHS or HFHS+EMPA for 18 weeks. Data are shown as mean ± SEM. Abbreviations as in Figures 1 and 2.


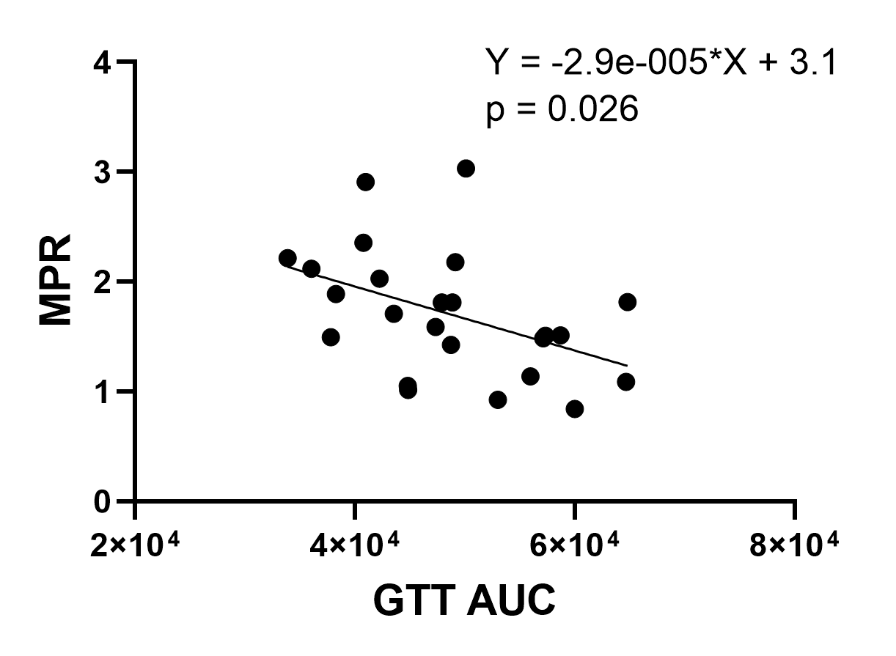


**Supplemental Figure 3. Relationship between glucose tolerance and myocardial perfusion reserve.** Linear regression plot between the AUC of the GTT curves and MPR for HFHS and HFHS+EMPA mice.

**
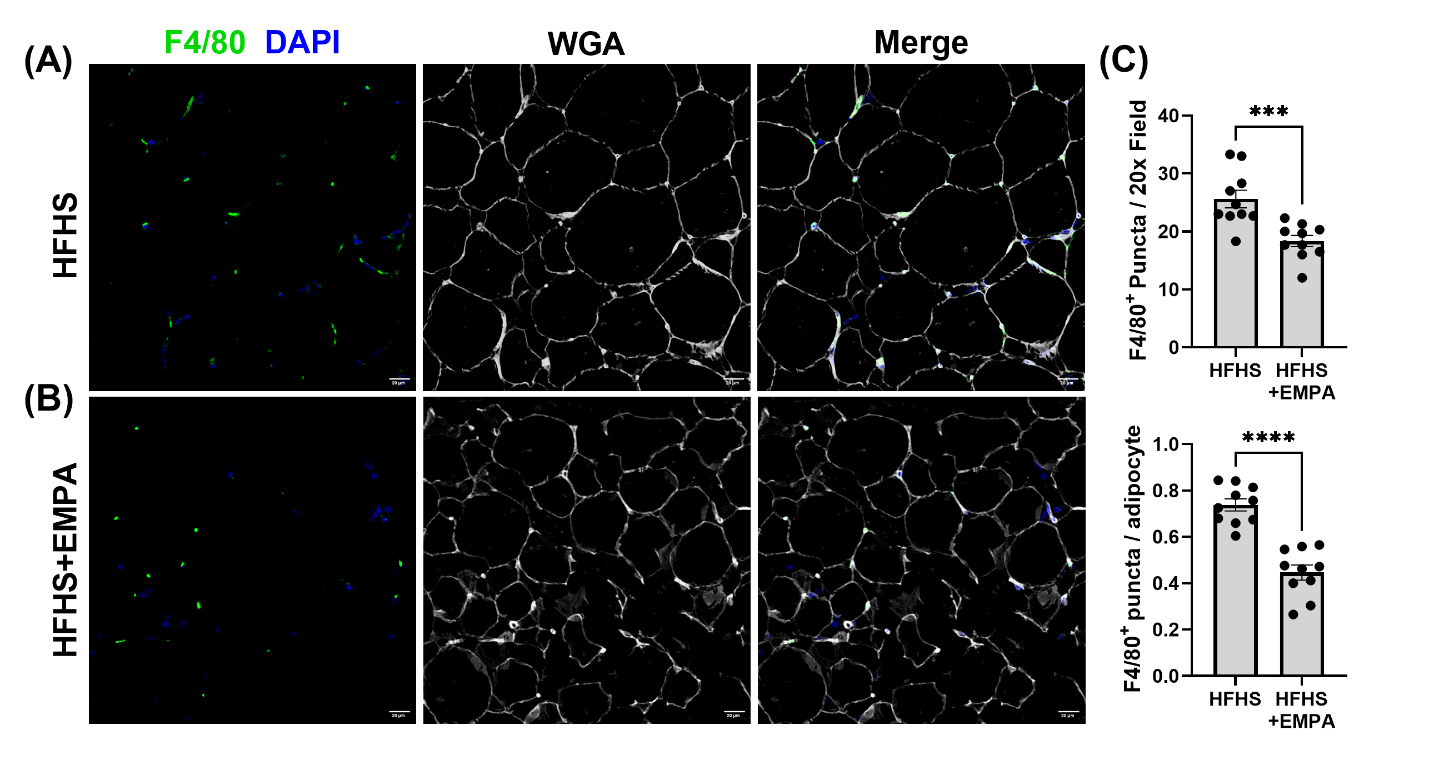
**

**Supplemental Figure 4. Effect of EMPA treatment on EAT macrophage infiltration.** Images of EAT from a mouse fed an (A) HFHS or (B) HFHS+EMPA for 18 weeks stained with DAPI (blue), F4/80 (green) for macrophage identification, and WGA (white) for adipocyte cell membrane identification. (C) Quantified F4/80^+^ puncta per 20X field and per adipocyte show reduced macrophage infiltration with EMPA (n=10/group). Data are shown as mean ± SEM and compared using a Mann-Whitney U test. Abbreviations as in Figures 1, 2, and 5.


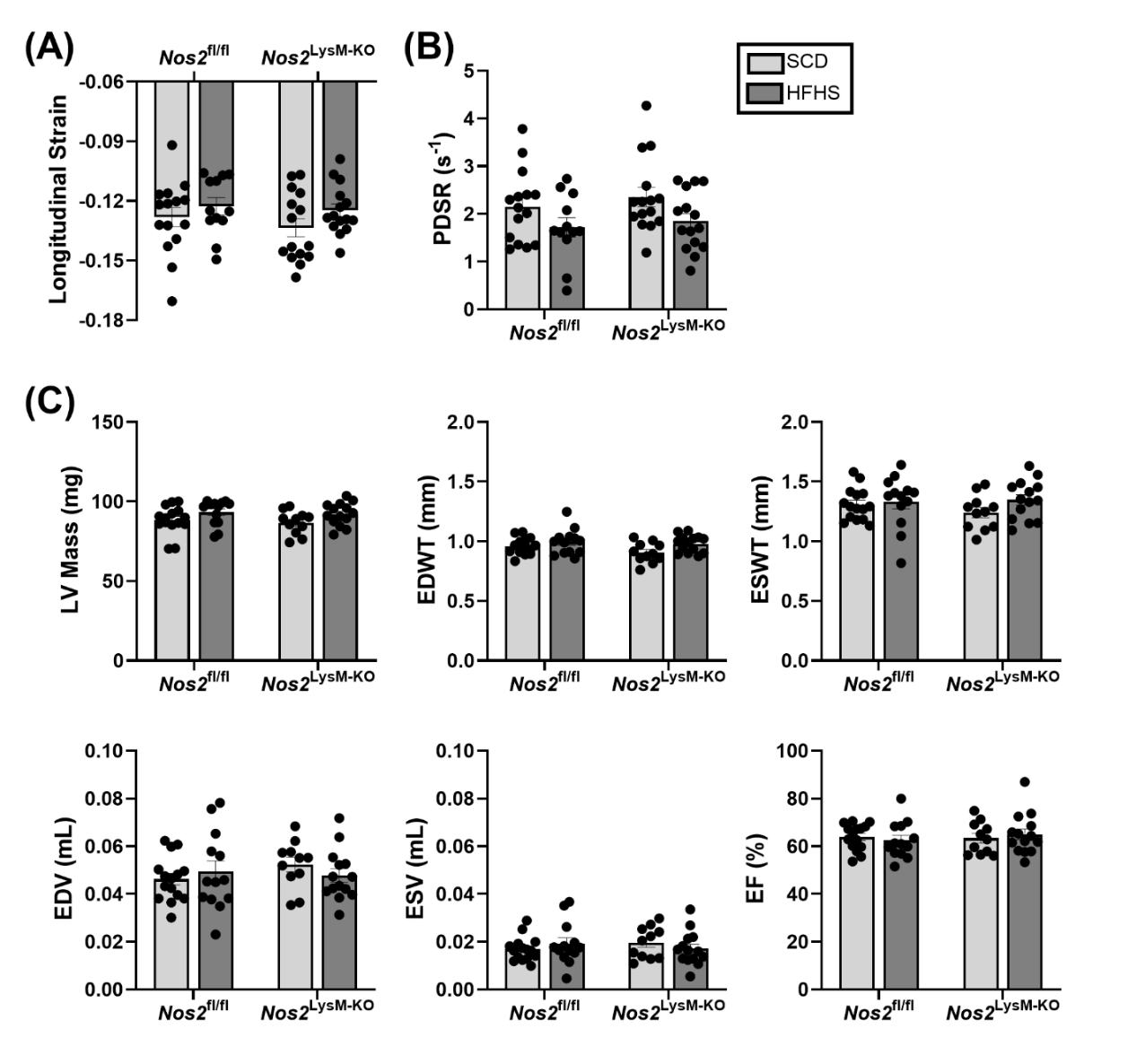


**Supplemental Figure 5.** **Effect of macrophage NOS2 on systolic strain, diastolic strain rate, and LV structure and function parameters.** (A) Global longitudinal strain and (B) PDSR for *Nos2*^fl/fl^ (SCD: n=15, HFHS: n=12) and *Nos2*^LysM-KO^ (SCD: n=15, HFHS: n=15) mice fed an HFHS diet or SCD for 18 weeks. (C) Cine-derived cardiac structure and function parameters including LV mass, EDWT, ESWT, EDV, ESV, and EF for *Nos2*^fl/fl^ (SCD: n=15, HFHS: n=13) and *Nos2*^LysM-KO^ (SCD: n=11, HFHS: n=14) mice fed an HFHS diet or SCD for 18 weeks. Data are shown as mean ± SEM and compared using an ordinary two-way ANOVA with Šidák’s multiple comparisons test.**P*<0.05 for indicated groups. All abbreviations as in Figures 1-4.

[1] Ackers-Johnson Matthew, Li Peter Yiqing, Holmes Andrew P., O’Brien Sian-Marie, Pavlovic Davor, Foo Roger S. A Simplified, Langendorff-Free Method for Concomitant Isolation of Viable Cardiac Myocytes and Nonmyocytes From the Adult Mouse Heart. Circulation Research 2016;119:909–20. https://doi.org/10.1161/CIRCRESAHA.116.309202.
